# Waiting and walking with strangers: a socio-psychological pedestrian experiment on joint action in anonymous situations

**DOI:** 10.1098/rsos.221601

**Published:** 2023-06-07

**Authors:** Krisztina Konya, Anna Sieben

**Affiliations:** ^1^ Civil Safety Research, Research Center Jülich, IAS-7, Wilhelm-Johnen-Straße, 52428 Jülich, Germany; ^2^ Department Social Theory and Social Psychology, Ruhr University Bochum, Universitätsstraße 150, 44801 Bochum, Germany; ^3^ University of St Gallen, Dufourstrasse 50, 9000 St Gallen, Switzerland

**Keywords:** pedestrian dynamics, crowd psychology, group behaviour, use of space, anonymity, mixed methods

## Abstract

Research on pedestrian dynamics has generally dealt with temporary gatherings of people who do not know each other personally. These gatherings are often framed as highly individualized encounters in which social interactions play no or only a marginal role. However, recent research based on self-categorization theory showed the relevance of salient social identity for crowd dynamics. Drawing on the interactionist approach of social identity theory and the work of Erving Goffman and Alfred Schütz, this paper aims to show that anonymous encounters are carefully concerted social phenomena. The authors present the results of an exploratory social psychological experiment (*N* = 83), in which groups of participants were asked to wait for 5 min in a designated area with different communicative conditions and then to walk to a narrow exit. Based on the assumption that communication and conformity to expectations influences the behaviour of those present, we introduced four modifications during the waiting time and analysed questionnaire data and video recordings in a mixed-methods design. The results show that direct communication correlates with higher speed, cell phone use with greater distance to the nearest neighbour, and unexpected behaviour with slower movement.

## 1. Introduction

Waiting and walking with strangers in public places such as train stations—this paper approaches these situations from the perspective of the interdisciplinary research field ‘pedestrian and crowd dynamics'. Broadly speaking, this research field investigates how large crowds occupy space and how people move in crowds, sometimes together and thereby creating collective phenomena [1–3]. To determine quantities such as density, velocity or acceleration of these movements, researchers rely predominantly on trajectories that are extracted either from empirical studies or from datasets resulting from simulations. Especially in recent years, the focus of these studies has been on modelling pedestrian flow and the prediction of pedestrian movements in various situations [2,4–6]. These models generally assume that individuals make rational choices which can thus be simulated just as one can predict the movements of particles due to the laws of physics. The models also consider the influence of spatial circumstances, as well as collective phenomena such as jamming, clogging in bottlenecks, stop-and-go waves in corridors, and lane formation in counterflow [7]. Furthermore, many physical pedestrian models treat other people mainly as ‘moving obstacles' and not as interaction partners. Paying attention to others is primarily a question of physical necessity.

In recent years, social psychological perspectives have become more integrated into pedestrian and crowd dynamics. In particular, applying the self-categorization theory (SCT) [8] or social identity theory (SIT) [9] to pedestrian dynamics has contributed to fruitful insights into the correlation between social affiliation and pedestrian dynamics [10–12]. Studies have shown that shared social identity, based on the recognition and awareness of membership in a group, changes walking behaviour [13] and counterflow patterns [14] in crowds: members of a crowd who are able to identify members of their own group (same coloured hat) maintain closer proximity to each other and walk significantly slower than members without such a group identity. One of the main categories developed by SCT is the binary difference between a physical crowd (a set of people who are co-present in the same space at the same time) and a psychological crowd (a co-present set of people who see each other as belonging to the same social category) [15]. Although this differentiation can be traced back to Le Bon's mass psychology, it takes on a fundamentally modified meaning within the framework of SCT: whereas Le Bon associated psychological unity in the crowd with the loss of rationality, orientation, moral concerns or critical faculties, SCT points towards processes of identification, affiliation and solidarity in psychological crowds. Undoubtedly, SCT and SIT can take the credit for shifting the focus from the individual to the relational view in crowds without following Le Bon's line, according to which the crowd embodies a collective irrational entity.

The scenario central to this article—people moving in a train station, waiting for the train and departing—falls precisely into the research field outlined above, but it also raises two additional sets of questions. First, research into pedestrian dynamics has rarely addressed waiting behaviour. To be more precise, waiting is generally considered less in terms of the use of space and more as a necessary disruption of movement (such as in bottlenecks or in front of closed entrances) and thus something to be avoided (as an exception, see [16]). This article, however, is interested precisely in waiting and considers it to be a space-use behaviour in its own right rather than a mere interruption. Second, we assume that even waiting and walking with strangers can trigger complex social processes that are not captured by the psychological versus physical crowd distinction. Following this distinction, a normal everyday situation at a city train station would be a typical example of a physical crowd [15]. However, we are interested in the twilight area between total strangeness and shared social identity in crowds that characterizes sharing public spaces with others. For this form of sociality, we use the term ‘anonymity': typically, in these situations, people unknown to each other share a confined space in close proximity with little intention of getting to know each other. It is our central assumption that people in these anonymous situations act as if they are not involved with each other, but that this very ‘casualness' is socially presuppositional and governed by subtle social processes. Furthermore, not all anonymous situations are alike, but the degree of bonding in those situations can be gradually distinguished. Sometimes people actually do verbally interact or feel close to each other for a brief moment without the anonymous framework breaking down. While previous social psychological work has primarily focused on psychological crowds, this article looks at so-called physical crowds with the intention of understanding and differentiating degrees of their sociality—a research approach which ultimately questions the very concept of ‘physical' crowds.

In order to understand these processes better, we have conducted an empirical study in which people unknown to each other waited and walked in an experimental set-up. We use three additional theoretical approaches to explore aspects of anonymity: first, the sociological work of Goffman; second, developments in SIT that focus on the communicative and interactive formation of social identity; and third, the phenomenological reflections of Schütz and Bernhard Waldenfels.

First, we draw on the comprehensive sociological work of Goffman [17–19], which served for decades as a basis for thinking about encounters in public spaces. Although he did not use the term anonymity himself, his detailed descriptions match our premise that waiting and walking with strangers constitute intensely socially concerted situations. Goffman described various types of proper and accepted behaviour in waiting situations, like respecting the personal space of others and simultaneously not positioning oneself so far away as to give the impression of being an outsider [17]. Particularly interesting are Goffman's reflections on the ‘civil inattention' that characterizes anonymous encounters and allows people to be in close physical proximity without giving the impression of invading the privacy of any others present [18]. Goffman describes in detail how focus needs to be finely managed in order not to appear too interested in others. This becomes particularly challenging when there is nothing to do aside from waiting for a train to arrive. In these situations, people like to find something with which to occupy themselves or which allows them to pretend at least that they are occupied. Leaning on Goffman's work, Collins [20] uses the term ‘interaction rituals' to emphasize the relevance of an appropriate emotional level in interactions which is constituted by shared emotional experience in co-presence. Preserving this shared atmosphere is part of managing the social ritual. However, according to this theory, shared emotional experience is not just distinctive for groups with high degrees of affiliation but is a component of any social gathering with the same attention focus. Following the considerations of Waldenfels regarding alterity and sociality [21], based on the main assumptions of phenomenology, we see in this regard similar reasoning in his differentiation between frontal and lateral sociality, meaning with the latter a social atmosphere which is transported by the shared attention directed towards a third party beyond the frontal connection between individuals [21]. Considering this, we could assume that the civil inattention, which Goffman ascribes to anonymous gatherings, has to be maintained by an affectless and indifferent atmosphere. In other words, anonymity is not an antisocial type of gathering but a special form of social relation which requires the maintenance of an atmosphere of indifference. These conceptualizations of anonymity serve, in this study, as the background for the qualitative video analysis of waiting behaviour.

Second, we turn towards one direction in social identity research which investigates the formation of identity rather than the influence of identity on behaviour. Postmes, Koudenburg and their colleagues [22,23] distinguish a deductive approach to identity (identity is treated as a given and resulting social behaviours are investigated) from an inductive approach (social behaviour is studied which leads to the formation of identity). The inductive approach directs the view towards those behaviours in groups or crowds of strangers which result in subtle changes of identity. It has been shown in smaller groups that this transformation towards shared identity can be affected by interaction and communication [22]. This work points towards blurred lines between emerging social states and emphasizes the highly performative character of sociality, at least in smaller groups [24,25]. For our work on anonymity, this opens up a space to think about the continuum between identity and anonymity. At first glance, anonymity and social identity seem to be mutually exclusive. However, we conceptualize them as two dimensions that are to some extent, though not completely, independent of each other. This acknowledges that one can build temporary bonds with strangers—without an attempt to explore this connection any deeper. Strangers on a platform can ignore each other or subtly acknowledge that the person whom they see every morning is back again, exchange unfriendly or friendly glances, or talk with each other briefly. Although all these situations maintain a certain degree of anonymity, there are still different atmospheres of being together and probably different levels of shared identity. At this point, it seems particularly fruitful to us to bring together the inductive approach of SIT [24,25] with Goffman's work. In fact, Goffman provides detailed descriptions of precisely those social mechanisms in groups in public spaces that inductive SIT lacks due to its previous focus on small groups. It addresses, namely, how people in public spaces interact and communicate to establish social connections with each other, or also to reject and prevent them.

Third, we also link the notion of anonymity to the concept of routine behaviour in everyday life. According to Schütz [26], the ‘lifeworld' offers space for action in everyday life on the one hand, and, on the other, is the summary of practical knowledge collected through everyday experiences. This store of knowledge, in the form of typifications, serves for managing situations. The grade of familiarity decides about the automatism of the interpretation of the procedure: the more familiar the situation, the less necessity to consider what will happen in the situation and which script to apply [26]. In this sense, anonymous situations in everyday life are familiar procedures in crowded and public spaces with automated response behaviour and a low degree of awareness about them. In other words, entering a social situation sets up certain expectations. If these expectations are met, there is no need for further interpretation. Diverging perceptions about the course of the interaction spoil the anonymous character of the situation and transform it into an event in which existing scripts are no longer sufficient or in which further interpretation is needed. This means that the degree of anonymity decreases if the behaviour of those present does not correspond to expectations.

To sum up, all three theoretical approaches indicate that anonymity in so-called physical crowds is not simply opposed to psychological crowds sharing a social identity. Instead, anonymity itself comes in different forms and shades and has to be socially performed. At least four aspects of anonymity can be derived from the literature:

(a) Focus: people need to perform being disinterested in others and occupied with something else (civil inattention).
(b) Atmosphere: on a more collective level they share an atmosphere of indifference.
(c) Social identity: in anonymous situation participants do not share a strong social identity; they do not know each other and they are not members of a distinct social group. However, anonymous situations can vary in the degree to which the concrete forms of communication and the joint movement lead to the formation of bonds between strangers.
(d) Scripts: congruence with expected scripts increases the anonymity of the situation.

In this paper, we introduce an explorative experimental study in which participants were asked to wait in a designated area. The participants did not know each other in advance. Afterwards, they walked towards a gate. Due to the COVID-19 restrictions in the year 2020, we were only able to conduct experiments with ‘small crowds' consisting of 7–12 participants. Communication, focus and congruency of expectations were systematically varied: in some cases, participants were allowed to speak with each other, which in others this was forbidden. Likewise, they were sometimes permitted to use their cell phones, and, in some experimental runs, a confederate acted in an unexpected, slightly awkward manner by talking very loudly on the phone about personal details. In a subsequent questionnaire, participants rated their perception of anonymity and social identity. Furthermore, participants were filmed from above, and we qualitatively observed their behaviour while waiting and recorded their exact trajectories while walking.

We expected a change in the perception of anonymity and social identity depending on the possibilities for interaction and on unfulfilled expectations regarding the situation and others' behaviour. Regarding the walking behaviour, we were expecting significant differences on the aggregate level of speed and distance of the participants to each other due to the forms of sociality (which was measured by a questionnaire study, see below). Our assumptions in detail were:

(1a) Verbal communication induces a higher grade of affiliation and shared social identity than no communication. We based this hypothesis on the interactive model of social identity formation (cf. [22,24,25]).
(1b) In line with the results of SCT studies, a higher grade of shared social identity may lead to slower speed and closer distances in comparison to lower degrees of social identity. This assumption is in line with the findings of Templeton *et al*. [13,14].
(2a) An individual and distracting waiting activity (cell phone use) impairs social perception (e.g. eye contact) and thus leads to a stronger feeling of anonymity than occurs without such an activity. The basis for this hypothesis is consideration of Goffman regarding maintaining indifference in order to be able not to communicate. (Goffman described that reading a newspaper served this need, which is an affordance in anonymous situations [17–19].) In this sense, we expect that using cell phones benefits the maintenance of the feeling of anonymity.
(2b) An individual and distracting waiting activity (cell phone use) leads to a particularly individualized behaviour when leaving the area (higher speed and bigger distances). This hypothesis aligns with hypothesis 1b.
(3a) Unexpected, awkward events reduce familiarity, as well as automatization, and lead to a higher awareness of the situation and thus a weaker sense of anonymity without increasing the degree of shared social identity. With this hypothesis, we lean on the considerations of Schütz. The interruption of everyday routines raises awareness of each other in the situation and therefore the feeling of anonymity decreases.
(3b) This awareness provokes a higher consciousness of others’ behaviour and thus similar walking patterns like cases of shared social identity (slower speed and closer distances).

## 2. Methods

### 2.1. Experimental setting

**Figure 1.**
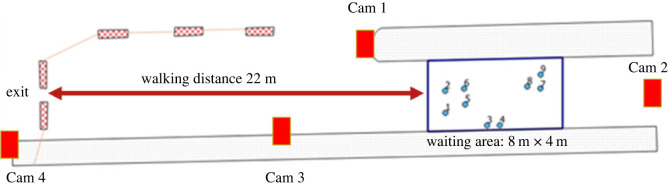
Experimental setting.

**Figure 2.**
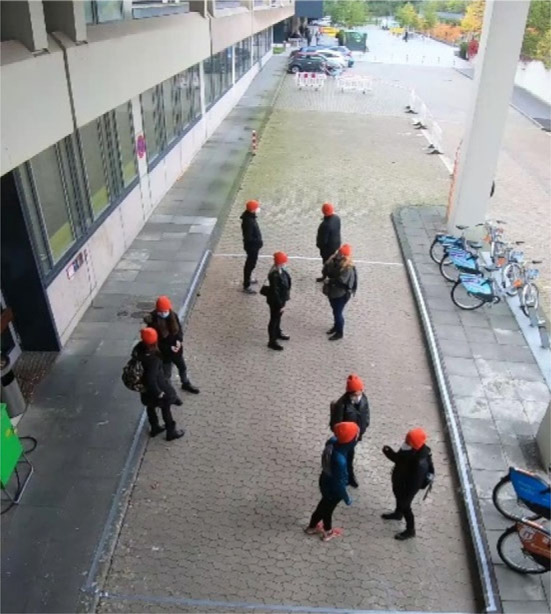
Waiting area from the back.

The experiment took place in October 2020 with the help of 83 participants (mainly students who received €10 for participating). The participants were divided into nine groups and invited to random time slots so that no social contact could occur before the experiment started. They did not know each other in advance, except for two pairs of friends who participated in the experiment in the same time slot despite the instructions in advance. After they had registered, the participants were led to the location of the experiment for a briefing. All groups received the following instructions: ‘Imagine you are in a train station concourse, and the marked area is a waiting area. Please go to this area now, you will wait there for a while. … [Further instructions depending on the specific condition, see below]… Thank you very much!' The participants entered the area (figure 1) and waited there for 5 min (figure 2 shows a screenshot of a group waiting). Subsequently, they were told that the experiment was over and that they should leave the area through the marked exit in order to fill out a questionnaire and get paid. We asked the participants in advance via e-mail to indicate if they would attend the experiments with friends, i.e. if they registered as a group. These participants were assigned to different timeslots to make sure that nobody in the distinct groups knew each other beforehand. Still, we could identify two dyads who seemed to know each other during the registration phase and asked them whether they knew each other. Because re-grouping was not possible at this stage of the experiments, they remained in one group. However, the video analysis showed that they did not behave differently from the others.

To modify the social experience while waiting, participants were variably asked to comply with one of following conditions:

1. Communicate verbally with the each other (While you are waiting, try to engage in conversation with the other people waiting.)—**‘speak’ condition**
2. Not to communicate verbally with each other (While you are waiting, avoid engaging in conversation with the other people waiting.)—**‘no-speak' condition**
3. Use their phones or find other distraction (While you are waiting, you are welcome to use your cell phone or other devices you have with you. Or read a book. If you don't have anything with you, you are welcome to get a crossword puzzle from me.)—**‘cell phone' condition**
4. Not to communicate with each other, while, however, a confederate made a phone call during almost the entire waiting time and pretended to speak to a representative of her telephone service provider. In doing so, she gave her name, address, date of birth and phone number, and did not lower her voice or turn away from the others during the call. The aim of this modification was to break the rule of anonymity in the crowd and at the same time create irritation because of the partial violation of the non-speaking rule of the crowd by the confederate.—**‘disturbance' condition**

Table 1 shows the distribution of participants according to the run, demography and experimental condition.

**Table 1.** Distribution of participants according to condition and run.

timeslot	female	av. age	student	condition	no. of participants by condition
timeslot 1 (*n* = 9)	67%	24	100%	**speak**	**26**
timeslot 4 (*n* = 10)	60%	24	100%		
timeslot 7 (*n* = 7)	29%	24	100%		
timeslot 2 (*n* = 10)	78%	27	90%	**no-speak**	**21**
timeslot 5 (*n* = 11)	64%	27	82%		
timeslot 3 (*n* = 7)	86%	23	100%	**cell phone**	**14**
timeslot 6 (*n* = 7)	43%	29	86%		
timeslot 8 (*n* = 12)	45%	26	90%	**disturbance**	**24**
timeslot 9 (*n* = 12)	50%	27	80%		

The entire area of the experiment was recorded from four perspectives by four cameras (marked in figure 1) with 4 K resolution.

The experimental procedure was approved by the ethics committee of the German Psychological Association (DGPs). Due to the COVID-19 pandemic in Germany at the time of the experiment, an outside area was chosen for safety reasons, participants and helpers were asked to disinfect their hands when they arrived and to wear a face mask for the entire duration of the experiment. All utensils (clipboards, pens etc.) were disinfected after each use. The participants maintained considerable distance from each other in every situation of the whole experiment including during the short waiting time to get registered. As social awareness was very high regarding the risk of COVID-19 infection at that time, we have to assume that social distancing would have been performed in a different way before the pandemic. However, the strict safety measures (FFP2 masks and outside environment) allowed for a relatively strong degree of freedom in moving with others. Furthermore, the COVID-19 context affected all four experimental conditions in the same way (without masks speaking would have been seen as riskier, but this effect was prevented by the masks). The possible impact of this specific global situation on the results is discussed in the conclusion section.

### 2.2. Mixed methods approach

Based on the assumption that behaviour in anonymous situations has a performative characteristic, we expected that the entire interactional process would prove important to understanding the behaviour of those involved. For this reason, we employed a mixed methods design: in addition to the questionnaire evaluation, which sought to capture participants' perceptions of social identity and anonymity, the behaviour of those waiting was analysed with the help of video recordings. The evaluation of the waiting behaviour was intended to capture subtle differences in the forms of sociality and to understand and connect behavioural aspects with the varying perceptions of sociality. The trajectory data were extracted and quantitatively analysed.

### 2.3. Video analysis

The evaluation was carried out using interpretative video analysis [27,28], applying sequential analysis and the hermeneutic approach, supplemented by common interpretations in interpretation groups. Interpretative video analysis is a method applied mostly in qualitative social research for analysing audio-visual records, whether they are commercials, field observations or experimental studies. Methodologically, it includes a hermeneutic approach: the main assumption is that ‘actions cannot merely be observed; rather, actions are guided by meanings' [28]. In this sense, the aim of the interpretative video analysis is the explication of subjective meaning in the observed action. Furthermore, subjective meaning is conceptualized as embedded in a cultural context which can be accessed by an ethnographic approach. Last but not least, the self-involvement of the researcher in the whole research process has to be considered, which makes the participation of several interpreters essential. In this framework, all nine runs were analysed in their full length, i.e. from arriving at the location of the experiment to leaving the location. A method of sequencing was applied, using sequences defined by the events which were relevant regarding for the behaviour of all participants in the run, such as listening during the instruction phase or positioning in the waiting area after the instruction. In the second step, the events or the behaviour of the subjects were described per sequence, whereby the focus in this phase was on precise and detailed description of overt behaviour with as little interpretation as possible. The third step took place after an interval of a few days. The video recordings and their textual description were appraised side by side to formulate an interpretation of the events based on the written description and the simultaneous viewing of the recordings. The second and third steps were complemented by co-interpretation sessions in interpretation groups with colleagues (2- to 4-person groups). In the fourth step, phenomena that seemed to be relevant were crystallized and described. Subsequently, all the material was re-screened and supplemented with regard to the phenomena observed.

### 2.4. Questionnaire

The questionnaire (see electronic supplementary material) consisted of 42 items with statements to be rated on a 7-point Likert scale (*1 = no agreement at all,* *7 =* *complete agreement*). The questions were divided into six sections: 1. Anonymity (three items), 2. Willingness to communicate/sociableness (three items), 3. Social identity (three items), 4. Perceived atmosphere while waiting (eight items), 5. Social norms during waiting and walking (14 items), 6. Overall perception during the experiment (five items). Since the dataset is rather small and sensitive to violation of the assumption of normal distribution, for the analysis we chose a non-parametric comparison method. First, the Kruskal–Wallis test was conducted for each item to determine whether significant group differences existed. If the *p*-value was less than 0.05, paired Wilcoxon–Mann–Whitney tests were carried out.

### 2.5. Trajectory evaluation

**Figure 3.**
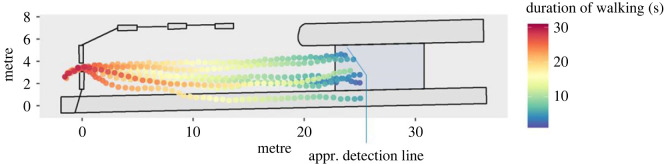
Trajectory coordinates during walking in the ‘cell phone' condition (third run). Coordinates of two frames per second for each participant. Duration of the whole walking period is illustrated from the moment when the instruction ended until the last participant left the area.

The recordings from Cam 1, a GoPro7.2 camera with a recording frequency of 25 frames per second, were used for the trajectory evaluation. The software ‘PeTrack' was used to extract the trajectories. This tool, which was developed by the Research Centre Jülich, makes it possible to determine the position of individual persons in each frame on the basis of colour differences in the recording (cf. [29]). Since all participants were asked to wear an orange hat and dark clothes, the contrast to the surrounding environment was sufficient to get very good results: the *x* and *y* coordinates could be determined in a calibrated coordinate system for each person and each individual frame (the footage contains 25 frames per second)^1^. For the trajectory evaluation, the area between the front side of the waiting area and the exit could be detected (figure 3).

**Figure 4.**
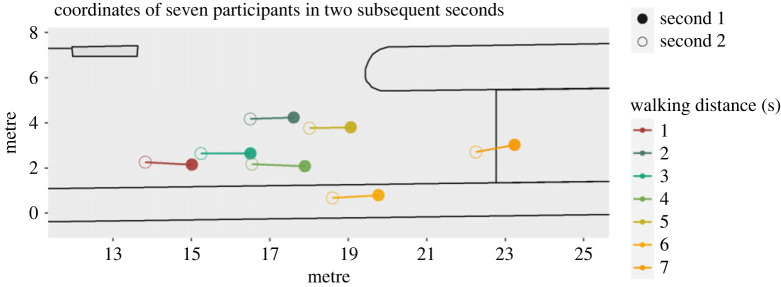
Calculation of speed (walking distance per second) in the third run.

Calculating the distance between the former and the actual position of individuals for each second results in the speed of the individual in m s^−1^ (figure 4).

**Figure 5.**
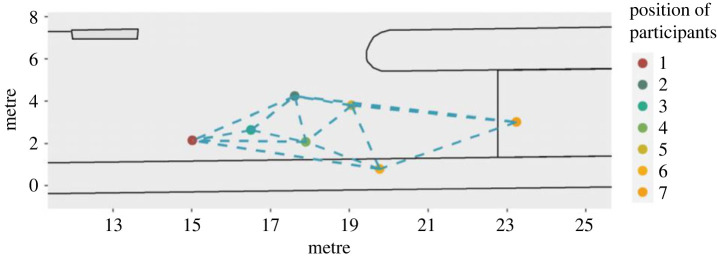
Delaunay triangulation after detecting all participants in the third run.

In the same way, calculating the Delaunay triangles between the coordinates of each participant in each second creates a triangle network in which all points are connected with their neighbours in a way that no other points can be included inside of each triangle (figure 5). In this way, it is possible to calculate the smallest distances in a crowd in a resource-saving way (instead of calculating the distances of all agents to all other agents) and then to select the shortest distance from this short set. Delaunay triangulation also forms the basis for calculating density in a crowd and is often used in pedestrian research (cf. [7]).

Because of the small numbers of participants per run, only the distance to the closest neighbour was calculated to avoid the distortion caused by outliers and different run sizes. The trajectories of five participants in total could not be included in the evaluation (three persons from the first run and two persons from the last run) because they had not understood the instruction at the end of the waiting period and consequently went to the exit only after a substantial delay. Two further participants in the fourth run asked a question after the general instructions were given, but this interruption caused only a small delay (approx. 2 s in comparison to the others), so the data for these persons were included in the analysis. All the other participants were included in the evaluation from the first moment in which the camera could detect all the participants of the run. For reasons of standardization, the 10 s intervals after detecting all participants of the run were used for the comparative analysis. Both the speed and the distance calculations refer to this standardization. The statistical evaluation was carried out with the statistical software R, v. 4.1.2, for the calculation of the Delaunay triangles and distances the package *spatstat* was used. We performed the same analysis like in case of the evaluation of the questionnaire: after proving the correlation with a Kruskal–Wallis test we conducted Wilcoxon–Mann–Whitney tests where applicable.

## 3. Results

### 3.1. Qualitative video analysis

#### 3.1.1. Use of space in anonymous situations: two phases of waiting behaviour

The analysis shed light on the relevance of the use of space while waiting, especially from the perspective of intersubjective ‘space management' and emphasized not only the temporal aspect of waiting but also the spatial. The video analysis clearly showed that the waiting for each group began at the moment when the instruction ended and they began entering the waiting area. The phase of positioning in the waiting area was clearly directed towards the time afterwards, which was anticipated to be a time of immobility. In the first phase, a place had to be found that served as a personal space reservation for the rest of the waiting time. After picking a spot and while waiting in the second phase, leaving the chosen place was generally not an option for the participants, regardless of the experimental conditions, and therefore rarely happened. Participants only changed their space for obvious reasons—participants also behaved in a way that justified their behaviour (for example, they walked away and sat down, very clearly communicating non-verbally that this position is more comfortable). This is necessary because moving away could otherwise be understood as a sign of social discomfort, as Goffman pointed out (cf. [18]). All participants seemed to be well aware of this immobility from the beginning of the positioning and accordingly, the positioning phase could be identified under each tested experimental condition as a phase of negotiating the spatial order and the waiting phase as maintaining the spatial order. These two phases fit into the behavioural observations of Goffman [17]: during the entire period of the experiment, participants strived to keep enough space to their neighbours and maintain equidistance to all their direct neighbours. This overall behavioural pattern, which can be connected to the attitude of indifference as a form of expression, has been complemented by the principle of chance: the participants endeavoured to give the impression of a completely accidental positioning.

#### 3.1.2. Positioning according to the anticipated sociality

The spatial order clearly varied depending on the expectations regarding the situation. According to the design of the experiment, the participants knew at the beginning of their positioning which communicational setting they would be confronted with while waiting (apart from one group, which only received the instruction ‘speak’ after the negotiation of the spatial order, in this case a new order was negotiated to be able to maintain the task). However, the formation also varied according to the size of the group.

**Figure 6.**
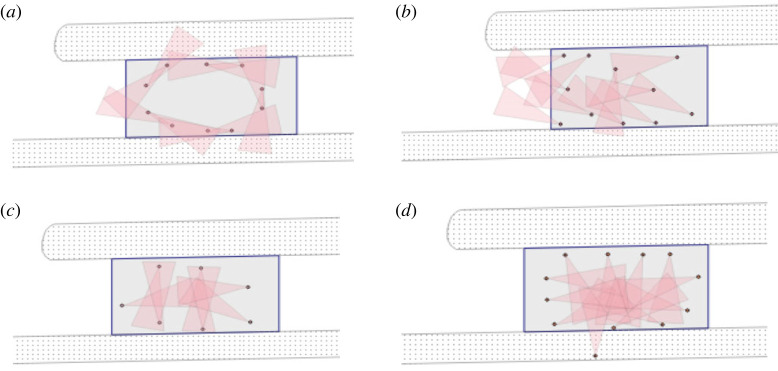
Typical positioning during waiting with view angle in the (*a*) ‘speak', (*b*) ‘no-speak', (*c*) ‘cell phone' and (*d*) ‘disturbance' conditions.

One test group of seven participants that was instructed to ‘speak' formed a small circle and conducted a group discussion that included everyone. The second test group that was given the same instruction included 10 participants who likewise initially formed a large circle. After the positioning process was completed, however, the neighbours turned to each other in groups of two and kept this dyadic order of interaction until the end of the waiting time (figure 6*a*). The third group accidentally received the instruction only after the positioning process and split up very fast into dyads and one three-person group. In all three of these experimental runs, other activities (looking around, turning around, looking at one's phone) were particularly rare; the conversation partners (whether in the small groups of two or in a large group of seven) focused on the conversation and thus looked at the people involved in the conversation. In all cases, the conversations were ongoing, and the participants were only interrupted in their conversation by the experimenter at the end of the waiting period.

The two test groups with instructions not to speak with each other fell into a formation in which the decisive direction of gaze was oriented towards the place of instruction, with individuals in the middle positioning themselves transversely, in the horizontal axis (figure 6*b*). As a result, some participants partially show their back to others. This backward direction can be interpreted as a body use in the interest of contact avoidance or rejection and as an expression of indifference. Additionally, avoiding the possibility for casual eye contact is also a strategy to not violate others' privacy (cf. [17]). Eye contact in public spaces can be interpreted as an appeal and request for a response (cf. [21]) and is therefore carefully managed.

The spatial arrangement under the condition of cell phone use could also provide indications of an existing connection between the physical turning away from others and the avoidance of possible eye contact. The members of both groups (each with seven participants) positioned themselves at equal distances along the edges of the waiting area, orienting their posture towards the centre of the room (figure 6*c*). Although not all participants followed the instructions by engaging in a pastime, about half of the people looked at their mobile phones during the waiting time. Table 2 shows the differences in the readable direction of gaze in both groups.

**Table 2.** Activity during waiting in the conditions ‘no-speak' and ‘cell phone’.

activity (measured the average duration in seconds per person)	no-speak	cell phone
using the cell phone	27.9 s	149.0 s
looking upwards	3.0 s	0.1 s
looking/turning to outside of the waiting area	70.0 s	22.4 s
walking around	27.7 s	8.6 s

The aggregated differences at the level of modification show that cell phone use clearly discouraged participants from looking around or walking around. This indicates that looking around and walking around can also serve as a kind of occupation for the waiting time. Furthermore, the spatial order directed towards the centre indicates that cell phone use can justify frontal positioning to others but also that others being distracted during the waiting time seems to liberate individuals from turning away. Interestingly, therefore, the cell phone groups look like they are turning towards each other, but in reality, this is due to a declared activity that offers a legitimate place to look and thus avoid accidental eye contact with others.

Although the ‘disturbance’ groups received the same instructions as the ‘no-speak' groups, both groups with this condition more distinctly assumed a geometric or circular position. The gaze of the participants, however, was not directed straight across to the other side of the area but to the left or right side, so the effort to evade eye contact could be observed in this condition, as well. In both groups, a person positioned themselves outside of the waiting area, which is likely related to the relatively high number of persons (*N* = 12) in these runs. In one group, two people spent almost the entire waiting time sitting on the edge of the pavement, busy with their cell phones, and the person closing the ‘circle' frequently turned around or away from the centre. Furthermore, this modification proved to be the most dynamic. In the first run, one participant, visibly annoyed by our confederate's phone call, left the enclosed waiting area and positioned himself next to the outside standing participant at a distance of about three metres against the wall. In the second group, another participant initiated a phone call after our confederate had continued to talk on her phone for some time. Similar incidents did not occur in any of the other groups.

In summary, the expected form of sociality had a distinct effect on the use of space during the waiting time. The knowledge of what kind of behaviour could be expected already impacted the positioning in the waiting area to make appropriate bodily practices like avoiding eye contact or participating in the interaction possible. Once the order had been negotiated with special care for equal distances between the participants, it was kept for the rest of the waiting time (with a few exceptions). Goffman describes this kind of immobility with the sensitive impacts of movement which can be interpreted like a social signal of rejection (in case of moving away) or attraction (in case of moving closer), thus a sort of expression of an affective connection to the regarding person (the neighbour). In this sense, all the test conditions can be considered as a sort of anonymous encounter with distinct rules of an affectless casual meeting. The differences between the groups can be characterized by the bodily practices which were used to perform the interactional situation.

### 3.2. Questionnaire study

#### 3.2.1. Perception while waiting

The aim of the survey was to explore differences in perceptions and expectations from different perspectives; therefore all items were tested individually for group differences. Table 3 shows the relevant items listed due to significant differences of one condition in relation to the other three conditions. Table S5 in the electronic supplementary material lists all items with results; electronic supplementary material, table S6, shows the test statistics for the items with significant differences.

**Table 3.** Relevant items regarding the differences between conditions. Level of significance: * *p* < 0.05, ** *p* < 0.01, *** *p* < 0.001.

significant differences in relation to	speak		no-speak		cell phone		disturbance		sign. diff. to all other conditions
	mean	s.d.	mean	s.d.	mean	s.d.	mean	s.d.	
*speak condition*									
**social identity measures**									
I felt a strong affiliation to the others present	4.54	1.58	2.67**	1.43	3.07*	1.82	2.5***	1.44	x
**sociableness**									
the people around me were sociable	5.42	1.58	2.19***	1.21	1.79***	0.89	2.14***	1.17	x
I was interested in getting in touch with the others	5.27	1.54	3.57*	2.29	3.07**	1.98	2.86***	1.58	x
I found the contact with others pleasant	5.54	1.58	4.19*	1.4	3.43**	1.55	3.68***	1.36	x
**anonymity**									
I felt anonymous while waiting	3.96	1.87	5.43*	1.43	5.21	1.63	5	1.83	
I had the feeling that the people around me wanted to be anonymous	3.12	1.48	4.71**	1.52	4.5	2.07	4.64**	1.4	
I would describe the people around me as an anonymous crowd while waiting	3.85	1.52	4.71	1.82	5.93**	1.21	5.36**	1.29	
**atmosphere**									
pleasant	4.85	1.74	3.33*	1.91	3.5*	1.22	2.86**	1.42	x
I let myself be influenced by the mood of the others	4.35	1.29	2.57**	1.75	3.43	1.7	3.23	2.05	
*fake condition*									
**certainty in behavioural norms**									
I was aware all along what was allowed	5.73*	1.56	5.62*	1.96	5.36	1.45	4.05	2.06	
**atmosphere**									
the overall atmosphere was pleasant	5.85**	1.26	5.38*	1.24	5.14	1.35	4.14	1.55	
I found the atmosphere unpleasant	2.15*	1.52	2.33	1.96	1.93*	1.21	3.14	1.52	
**overall perception**									
I found it pleasant to stand in the waiting area	4.85*	1.76	4.86*	1.8	4.57	1.99	3.36	1.84	
*cell phone condition*									
**behaviour of others**									
the others around me were social distancing	5.73*	1.61	6.57	0.75	6.79	0.43	5.18*	1.92	

The question block about the behavioural rules turned out to be irrelevant in this context except as regards one question. Although members of all the groups agreed to some extent with the statement *the others around me were social distancing*, those in the group instructed to use their phones gave the statement a noticeably higher rating.

Our hypothesis that speaking with each other would lead to a higher grade of social identification than other conditions (hypothesis 1a) can be confirmed for the aspect *feeling strong affiliation* to others present in this condition which generated a significantly higher agreement in comparison to all other conditions with a strong effect size of *r* = 0.48 and *r* = 0.49 in the case of no-speak and disturbance and a medium effect size of *r* = 0.34 in the cell phone condition. It is hardly surprising that the aspects of similarity (*I identified myself with the others present in the situation*, *I am like the others in the situation*) did not show distinct differences (these items are therefore only listed in electronic supplementary material, table S5), since most of the participants were students at the same university and were well aware of this fact. Additionally, the experimental setting can also generate a common ground based on mutual experience (cf. [23]). This mutual experience might also explain why answers to the item ‘feeling a strong affiliation’ in the conditions no-speak, cell phone, and disturbance were lower than in the speak condition (see above) but still higher than 1 (the lowest possible number). In this sense, the results show that participants recognized their membership in the same social category, but that the interaction during the waiting time still made a significant difference for feeling affiliated. This result corresponds with our expectations.

According to hypothesis 2a, the use of cell phones would lead to a stronger perception of anonymity than under the other conditions. This assumption could be verified when compared to the ‘speak' condition (MD = 6 compared to MD = 4 and an effect size of *r* = 0.51) regarding the overall evaluation of the situation as an anonymous situation (*anonymous crowd during waiting*). Even though this statement generated stronger agreement in the cell phone group than in the other groups, the other two anonymity items did not display similarly strong differences. The weaker perception of anonymity on the individual level (*I felt anonymous*, *I have the feeling that the others wanted to remain anonymous*) in the condition ‘cell phone’ might be due to the distraction. As the results of the video analysis have already shown, cell phone use was generally related to a lower observation of the surroundings. This does not preclude, however, an even stronger perception of an anonymous atmosphere. This could also explain the significantly higher rate of agreement with these two statements by the participants in the ‘no-speak’ condition: a high (i.e. not distracted) awareness of not being in contact with each other and still having to deal with the obvious presence of the others with a lack of distraction may raise the perception of anonymity at the individual level stronger than regarding the overall perception in the situation. The results therefore show that the perception of anonymity can be affected by different types of communicational setting.

Not only cell phone use but also the unexpected phone call (disturbance) led, however, to a distinct difference (MD = 5.5 versus MD = 4 with an effect size of *r* = 0.45) in the overall evaluation of the level of anonymity from the groups under the ‘speak' condition, with only a slight difference from the ‘cell phone' runs. This result contradicts the expectations formulated in hypothesis 3a. In addition, regarding the aspect of the willingness to give up anonymity by the others, the ‘disturbance' condition differs significantly from the ‘speak' condition (MD = 4 versus MD = 3, *r* = 0.43), suggesting that not even our confederate's sharing of personal information affected the perception of anonymity in a similar way to the case of interaction. These differences can also be explained in part by atmospheric disparities. Especially in comparison to the ‘speak’ condition, participants reported an unpleasant and less agreeable atmosphere (MD = 2 versus MD = 3, *r* = 0.26). The item *while we were waiting, the atmosphere was strained* just slightly failed to reach the significance level in comparison to the ‘speak' condition. As reported above, the experimenter noted a palpable level of annoyance and impatience. These discrepancies refer to the high relevance of atmosphere for anonymity and the perceived sociality in these situations. Last but not least, a relevant difference could be shown regarding participants' certainty of social norms: participants in this condition were more uncertain about what was allowed in the situation (in comparison to the speak and no-speak conditions in both cases MD = 6 versus MD = 4 and a medium effect size of *r* = 0.34). These results lead us to conclude that the effect of uncertainty combined with a rather unpleasant atmosphere contributed to even stronger feelings of anonymity, in contradiction to our expectation. However, the ‘disturbance' condition does display a high impact of atmospheric distinctions and obvious differences in the perception of sociality during the waiting time.

### 3.3. Trajectory analysis

**Figure 7.**
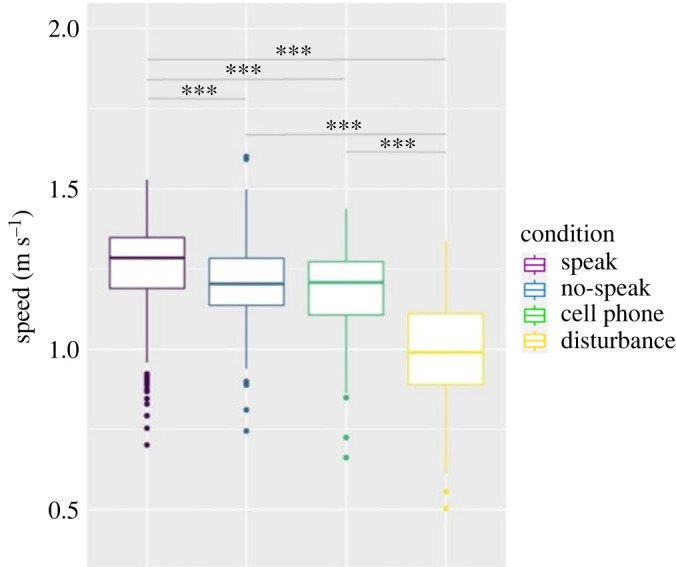
Average speed over all runs per condition (m s^−1^), reference to the standardized walking time, median and standard error. Level of significance: * *p* < 0.05, ** *p* < 0.01, *** *p* < 0.001.

**Figure 8.**
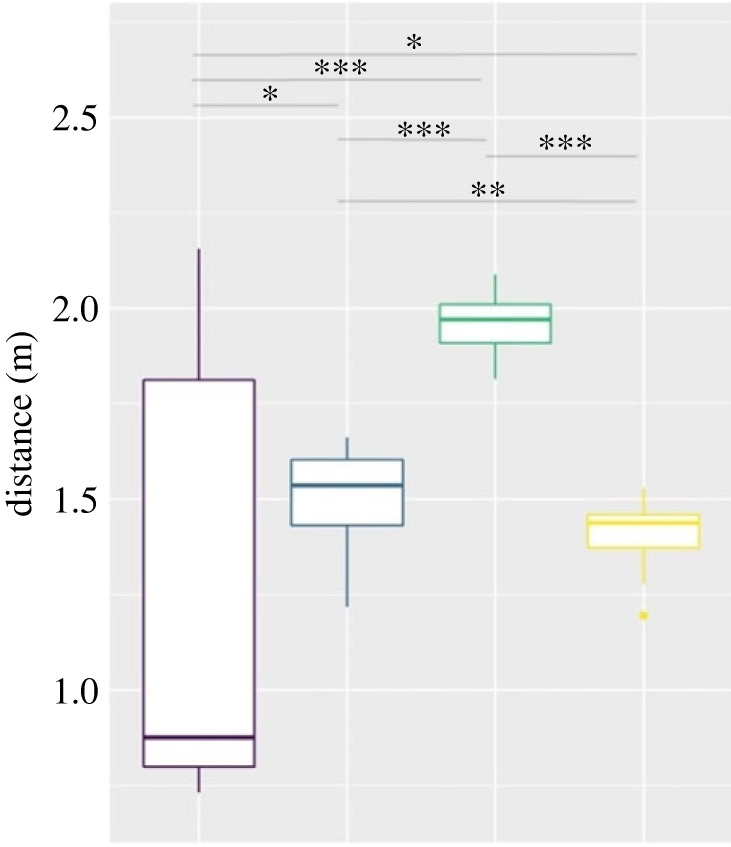
Average distance to the closest neighbour over all runs per condition, reference to the standardized walking time, median and s.e. Level of significance: * *p* < 0.05, ** *p* < 0.01, *** *p* < 0.001.

We expected significant differences regarding the average speed of the participants and the average distance to the closest neighbour of each participant in a run (see Methods section for description of trajectory analysis). Figures 7 and 8 show the medians and standard errors per condition with the level of significance in between conditions. Table 4 in appendix A shows the exact values per condition. As indicated in the Methods, the comparison was based in this case on the paired Wilcoxon–Mann–Whitney test, as well.

This first part of hypothesis 1b, due to which participants in the ‘speak' condition were supposed to walk with significantly lower speeds in comparison to other conditions, could not be confirmed. In fact, the result is the opposite of what we expected. After speaking with the others present, the participants walked significantly more quickly to the exit than those in all the other conditions. The explanation for this result can be found in the interaction itself: communication with one partner enabled participants to refer exclusively to the movement of their conversational partner. This is particularly true for two of the three runs in this condition: the dyadic structure was maintained as they walked, and the participants continued to speak with each other the entire time except when interrupted by the instructions. In the case of the group conversation in the third run of the ‘speak' condition, the participants were not able to communicate in a circle while walking and refrained from further conversation. However, the participants tried to maintain contact to the other participants by enabling a free visual angle to everybody in the run and thus expressing a willingness to act as a group. As a result, this group retained the circular shape while walking to the exit. Interestingly, this difference did not impact the speed; the circular ‘speak' run was even slightly faster than the other two runs (MD_run1 = 1.23 m s^−1^; MD_run4 = 1.25; MD_run7 = 1.28 m s^−1^).

**Figure 9.**
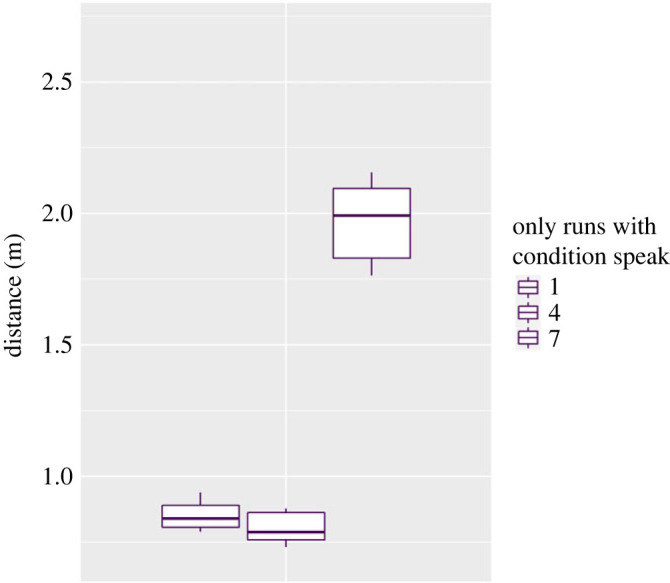
Median and s.e. of the runs with condition ‘speak' (exact values in table 4 in appendix A).

On the other hand, the second part of hypothesis 1b, which expected participants in the ‘speak' condition to walk in closer proximity to each other, could be partially proved. As the first boxplot in figure 8 indicates, there was a high variation in proximity in this condition. However, the wide range of the standard error can be traced back completely to the different walking pattern in the third run of the ‘speak' condition; without this run the distances to the closest neighbour averaged MD = 0.83 m (s.e. = 0.013) in the ‘speak’ condition (figure 9). This latter short distance could be explained by the circumstances of the conversation on those so engaged: being able to understand each other requires proximity.

It is worth mentioning that participants walked closer than 1.5 m to each other—which was at that time the required distancing due to COVID-19. This might be an indicator of participants feeling safe in the experiments and therefore showing walking patterns that might be comparable with a non-pandemic situation.

Considering the ‘disturbance' condition, the differences regarding both speed and social distancing seem to satisfy our expectations. While the speed averages for the ‘speak', ‘no-speak' and ‘cell phone' conditions move between MD = 1.18 m s^−1^ and MD = 1.25 m s^−1^, the walking speed in the ‘disturbance' condition with MD = 0.99 m s^−1^ is clearly slower. We can thus confirm our hypothesis 3b that a disturbing event and the uncertainty about the procedure makes the participants slow down and heightens awareness (i.e. cautiousness) of the movement of the others present. We could not find this distinct difference regarding the closest distances during walking; still the relatively small difference of 20 cm from the ‘speak' condition seems to support our expectations.

Hypothesis 2b could only be confirmed with regard to the maintained distances of the participants to each other, but not with regard to the speed differences. The distraction of cell phone use did not cause a higher speed in comparison to the ‘speak' and ‘no-speak' conditions, but it made a distinct difference regarding social distancing during walking. With MD = 1.97 m, participants in this condition remained at least half a metre further away from their neighbours than in any other condition. It may have to do with the fact that some participants continued to play with their mobile phones while walking and supports the assumption that the waiting time and subsequent use of space cannot be considered completely separate from each other.

## 4. Discussion

Summarizing our findings, we see our main assertions supported: different modes of communication create different forms of perceived sociality and the perception of sociality correlates with different walking patterns. Furthermore, the results show that expectations regarding social behaviour in a certain situation affect the use of space and self-organization in a crowd. This latter finding is mostly based on the video analysis of the waiting behaviour, which showed that participants used their bodies to promote the anticipated mode of interaction, depending on how communicative they were instructed to be. In the case of the ‘no-speak' condition, this meant the prevention of any type of non-verbal contact, including eye contact. This physical prevention from communication was less pronounced when participants were distracted by cell phone use; this may be attributed to the expectation that eye contact can be easily avoided by looking on the cell phone. Participants with the instruction to speak with each other chose a dyadic form of interaction in two runs, while in the third run all participants took part in one conversation.

Furthermore, qualitative video observation revealed two distinct phases of waiting. In the first phase, spatial order is negotiated and participants take a position (not too far away, not too close, equidistance is sought). In the second phase, participants stay in place—this immobility can only be broken if behaviour can nonverbally communicate a reason for the shift in place (e.g. someone goes to look at the information board). Otherwise, a shift in place in the second phase can be understood by bystanders as someone intentionally decreasing or increasing the distance to them. This is generally avoided in anonymous situations.

The differences between the experimental conditions could be tracked in the usage of space during the waiting time, but also in the walking formation as participants left the waiting area. Regarding the latter aspect, in contradiction to our expectations, we see slightly higher speed in runs in which participants spoke with each other than under all other conditions. One explanation of this result could be the difference in the necessary attention to the movement of the surrounding individuals: being in a conversation can liberate participants from paying attention to the movement of all the others and in this way support a faster walking speed. However, we also have to consider that the differences especially in comparison to the no-speak and cell phone conditions are rather small, even if statistically significant.

We found a slower speed in the ‘disturbance' condition in which our confederate answered a phone call despite the instruction to refrain from speaking with each other during the waiting time; this finding is in line with our hypothesis. However, we expected this behaviour to foster a less anonymous atmosphere due to the mutual attention to the disturbing event and others' reaction. We could indeed measure significantly higher insecurity in this condition regarding the behavioural norms and an atmosphere perceived as less pleasant, but the phone call was not connected to a weaker perception of anonymity. On the contrary, the irritation seems to support a more alienating experience, but also a higher level of attention. Our assumptions that encouraging participants to use their cell phones while waiting would raise the perception of anonymity and increase the speed and distance of the participants to each other could be confirmed in both respects.

As an additional precautionary remark, we have to take into account that the measured speed differences due to the modifications, especially the differences between the speak respectively no-speak and cell phone conditions in real life can be considered as common deviations. In this study, we did not have the opportunity to test our hypotheses with a larger number of runs, which would have been necessary to provide more reliable results. The small number of cases, the small number of runs and the homogeneous composition of the participants (mainly students) can be seen as clear limitations of this study which have to be considered when interpreting the results.

## 5. Conclusion

Here, we conclude that situations like leaving a place together with strangers show more socially coordinated joint action than we normally assume. Looking back, estimating where the others plan to walk, if they may speed up, or if it is necessary (and polite) to walk slower to avoid impinging on others' personal space—these strategies and considerations have been observed in the video analysis in all groups. These considerations happened, however, in a rather indirect way (still avoiding direct eye contact, turning only halfway in the direction of others etc.). A direct reference to persons and an open adaptation to others' movements are not options which can be carried out in anonymous crowds. Conversations, however, enable participants to refrain from showing indifference and acting as if these adjustments occur by ‘chance' and to turn directly towards strangers. In this sense, conversation can loosen up anonymity and, interestingly, contribute to a liberated (from the whole group) locomotion (which is in our experiment also slightly speedier).

Furthermore, the results show that it is not only positively connoted social affiliation that affects cohesive locomotion but also unsettling (albeit not necessarily threatening; cf. research on the cohesive effect of disasters [30,31]) situational social disorder. In this respect, mutual expectations for the social setting of the situation have to be considered in detail to achieve a better understanding of pedestrian dynamics in public spaces. Very few studies [32,33] have considered the integration of the anticipation of others' behaviour into one's own walking behaviour. As Murakami *et al*. [34] recently showed, visually distracting just a few pedestrians in a crowd, and thus complicating their mutual anticipation of behaviour, can significantly impede the self-organization in a bidirectional flow. Our study indicates that not only the anticipation of individual motion, but also the anticipation of sociality (in terms of communicative conditions) and social coordination (in terms of appropriate behaviour) is a crucial aspect of pedestrian dynamics.

In comparison to other crowd psychology studies (cf. [13,14]), identity was not constructed on the basis of a binary difference between in- and out-groups, but developed in the experiment as a performative type of social identity (cf. [22]). This difference might explain the deviation from the findings of the studies of Templeton *et al*. (which showed slower speed for groups sharing identity, while we saw slightly higher speed) and highlights the necessity of taking a closer look at the performative character of anonymous situations to differentiate between forms of social identity with regard to pedestrian dynamics: in anonymous situations the emergence of affiliation to others is not necessarily accompanied with walking together closely as a large group. Instead, we observed the spontaneous formation of pairs in the group which, in a way, liberated the movement from the group—except for the conversational partner.

More generally, on the basis of our study, it seems to us particularly promising to focus in the future on inductive processes of the formation of identity and social norms [23] in crowds and thereby to expand this social psychological approach from small groups to large groups. Research to date has tended to consider social psychological perspectives in regard to pedestrian dynamics primarily with an interest in how pre-existing identities, affiliations, or norms affect the concrete dynamics. It is at least as interesting to ask how waiting and walking together set dynamics in motion that result in new forms of identity, other norms, or different arrangements of anonymity. Integrating the micro-sociological framework of Goffman enables one to focus on the situational conditions as well as on the performances of anonymity and/or identity in public spaces via posture, body movement, or attention focus. Involving routine behaviour in everyday situations based on the phenomenological sociology showed the differentiating effect of social expectations. Conceptually, these reflections question the ‘physical' part in the psychological versus physical crowd distinction. Clearly, anonymous crowds without a shared social identity are not a-psychological, but rather psychological in a different way. Further theoretical work is needed in order to present a new typology of crowds which dispenses with the concept of the physical crowd without losing the helpful conceptual distinctions between different kinds of crowd introduced by SCT.

In this work, we could not consider all aspects of anonymity which could be relevant for pedestrian research. However, our results show how the perception of anonymity is linked with waiting and walking behaviour. Further conceptual and empirical work is needed in order to develop anonymity as a fruitful concept. Used in the common sense, anonymity means mostly being unknown to others or being alone in a big city. However, as the mentioned theoretical works testify, anonymity can be considered as an overarching concept with various aspects, like atmosphere and attentional focus. We argue that anonymity is not the same as individuality: although people might feel ‘on their own' in an anonymous situation they do not necessarily feel they are distinct or ‘stand out' as the term individuality suggests. Furthermore, not knowing anything about the others does not lead necessarily to a strong perception of being individuated or to an individualizing behaviour. However, further aspects like the attentional focus can trigger these factors, or conversation can moderate them. Following these considerations, we argue that anonymous situations should be considered as social (and not individuated) encounters with further differentiations regarding the type of the situation. This approach could contribute to a more detailed and multifaceted picture of social dynamics in crowds, without neglecting the importance of shared and performatively emerging social identities.

Furthermore, future empirical work is needed in order to fully integrate large crowds: the experiments analysed here were restricted by the COVID-19 pandemic. On the one hand, we conclude that the results were not strongly influenced by the pandemic. The safety measures allowed participants to move relatively unrestricted—indicated by the fact that they undercut the prescribed distance of 1.5 m in Germany at that time. On the other hand, the sample size had to be drastically compromised due to the pandemic and therefore similar experiments with more participants in each group should be conducted in the future. Like all laboratory experiments, ecological validity is limited. Comparisons with fieldwork data, for example from train stations, would be instructive. However, these situations are usually not observable to the same extent due to data protection issues. Potential solutions include the use of recordings of trajectory data only (without video footage) such as analysed in [16]—but these lack the details needed for a social psychological study (subjective data, video footage with details such as focus, posture, body orientation). However, a systematic combination of both approaches might be a future avenue to increase ecological validity.

This study is interdisciplinary, but also uses mixed methods research design. The combination of trajectory evaluation and qualitative and quantitative social research methods has thus far been very rare in the field of pedestrian dynamics, with a few exceptions [13,34]. Only some studies supplement their analysis with qualitative observations of the experimental situation (cf. [35]). However, a systematic and methodically funded qualitative approach from the first research phase onwards can improve the overall explanatory quality of the study, enable a detailed and in-depth description of social phenomena, and support the emergence of new insights beyond the mainly binary hypothesis testing of a merely quantitative experimental setting.

This is directly related to a general methodological question regarding socio-psychological experiments in the field of crowd and pedestrian dynamics. For every experimental run, new ‘crowds' are needed—easily resulting in unrealistic sample sizes. In the study presented here, we were additionally limited by the condition of the COVID-19 pandemic and the difficulty of recruiting large samples. Furthermore, in experiments, researchers have the possibility to initiate certain forms of behaviour, but people in the crowd also influence each other strongly. The larger the crowd, the higher the probability that the unexpected behaviour of individuals will change the entire experiment. Similarly, even in smaller scale experiments, repetitions of the same experimental condition might result in different findings because individuals in the crowds have initiated quite different social dynamics. We have seen this in the case of the three ‘speak' runs. Therefore, exact experimental repetition of sensitive social constellations in large crowds will probably never succeed. For us, an in-depth qualitative analysis is therefore crucial not only to understand the different outcomes of experimental runs in retrospect, but also in order to validate the underlying assumptions of the study, as a kind of manipulation check. As we saw, even in the case of different outputs, a qualitative analysis can ensure that the main assumptions of the study still apply. Rather than seeing deviations of runs within the same experimental condition as a methodological weakness, we argue for a mixed-methods approach which takes a closer look at the details of the social conditions and unplanned circumstances of the individual runs and draws inspiration from the observed (ir)regularity of social events. In this way, one can also account for the unexpected socio-psychological dynamics of crowds.

As final remark, we reflect on applying our results to modelling of pedestrian dynamics. This study shows that pedestrian behaviour is embedded in a social context that is framed by expectations towards the situation and by behavioural rules which are obligatory on a rather non-reflected way. For one situation—waiting on a ‘platform' and walking towards a bottleneck—not just one behavioural rule is available but several, and their activation depends on situational social factors such as communication. The challenge for models is to incorporate the alternation of different rules of behaviour. While most models assume that the social formations (e.g. small groups; see [11] for a review on how small groups are modelled) and the rules of behaviour (e.g. cooperative versus competitive behaviour; see [36] for an overview) are predefined, in our experiments the rules of behaviour are chosen in the situation. To model such context-dependent decisions for certain behaviours, Seitz *et al.* [37] propose a model that uses cognitive heuristics. Similarly, it might be possible to model different types of social contexts in our experiments. However, in order to formulate a well-defined set of social–cognitive heuristics, it is necessary to repeat the experiments with larger groups as described above. Here, the precision required by models can help to precisely delineate the different behaviours observed. In this sense, closer cooperation between social psychology and modelling can contribute to a better understanding of social dynamics in pedestrian crowds.

## Data Availability

The data used to support the findings of the paper are available on the following website: https://ped.fz-juelich.de/da/doku.php?id=waiting_with_strangers (https://doi.org/10.34735/ped.2020.2) [38]. Supplementary material is available online [39].

## Footnotes

1 The calculation was based on an average height of 180 cm for each participant. Our confederate completed all tasks of the experiment for credibility. Since an exclusion would have distorted the distance calculations, her trajectory data are part of the calculation. Her questionnaire data were excluded from the analysis.

## Appendix A

**Table 4.** Mean, standard error and median of speed and distance to the nearest neighbour. The third part of the table shows the values without run 7.

condition	speed			distance			distance without run 7		
	mean	s.e.	median	mean	s.e.	median	mean	s.e.	median
*speak*	1.25	0.01	1.28	1.21	0.10	0.88	0.83	0.01	0.82
*no-speak*	1.21	0.01	1.20	1.50	0.03	1.54	1.50	0.03	1.54
*cell phone*	1.18	0.01	1.21	1.97	0.02	1.97	1.97	0.02	1.97
*disturbance*	0.99	0.01	0.99	1.41	0.02	1.44	1.41	0.02	1.44

## References

[1] Moussaid M, Garnier S, Theraulaz G, Helbing D. 2009 Collective information processing and pattern formation in swarms, flocks, and crowds. Topics Cogn. Sci. **1**, 469-497. (10.1111/j.1756-8765.2009.01028.x) 25164997

[2] Vizzari G, Manenti L, Ohtsuka K, Shimura K. 2015 An agent-based pedestrian and group dynamics model applied to experimental and real-world scenarios. J. Intell. Transp. Syst. **19**, 32-45. (10.1080/15472450.2013.856718)

[3] Sieben A, Schumann J, Seyfried A. 2017 Collective phenomena in crowds—where pedestrian dynamics need social psychology. PLoS ONE **12**, e0177328. (10.1371/journal.pone.0177328) 28591142 PMC5462364

[4] Gibelli L, Bellomo N. 2018 Crowd dynamics, volume 1. Modeling and simulation in science, engineering and technology. Cham, Switzerland: Springer International Publishing.

[5] Ronchi E, Uriz FN, Criel X, Reilly P. 2016 Modelling large-scale evacuation of music festivals. Case Stud. Fire Safety **5**, 11-19. (10.1016/j.csfs.2015.12.002)

[6] Bailo R, Carrillo JA, Degond P. 2018 Pedestrian models based on rational behaviour. In Crowd dynamics, vol. 1 (eds L Gibelli, N Bellomo), pp. 259-292. Cham, Switzerland: Springer International Publishing.

[7] Schadschneider A, Chraibi M, Seyfried A, Tordeux A, Zhang J. 2018 Pedestrian dynamics: from empirical results to modeling. In Crowd dynamics, vol. 1 (ed. T Gibelli), pp. 63-102. Berlin, Germany: Springer International Publishing.

[8] Turner JC. 1988 Rediscovering the social group. A self-categorization theory. Oxford, UK: Blackwell.

[9] Tajfel H, Turner J. 1979 An integrative theory of intergroup relations. In The social psychology of intergroup relations (ed. WG Auston), pp. 33-47. Monterey, CA: Brooks/Cole.

[10] Neville FG, Novelli D, Drury J, Reicher SD. 2022 Shared social identity transforms social relations in imaginary crowds. Group Process. Intergroup Relations **25**, 158-173. (10.1177/1368430220936759)

[11] Templeton A, Drury J, Philippides A. 2015 From mindless masses to small groups: conceptualizing collective behavior in crowd modeling. Rev. Gen. Psychol. **19**, 215-229. (10.1037/gpr0000032) 26388685 PMC4568938

[12] Drury J, Cocking C, Reicher S, Burton A, Schofield D, Hardwick A, Graham D, Langston P. 2009 Cooperation versus competition in a mass emergency evacuation. A new laboratory simulation and a new theoretical model. Behav. Res. Methods **41**, 957-970. (10.3758/BRM.41.3.957) 19587213

[13] Templeton A, Drury J, Philippides A. 2018 Walking together. Behavioural signatures of psychological crowds. R. Soc. Open Sci. **5**, 180172. (10.1098/rsos.180172) 30109073 PMC6083654

[14] Templeton A, Drury J, Philippides A. 2019 Placing large group relations into pedestrian dynamics: psychological crowds in counterflow. Coll. Dyn. **4**, 1-22. (10.17815/CD.2019.23)

[15] Reicher S. 2011 Mass action and mundane reality. An argument for putting crowd analysis at the centre of the social sciences. Contemp. Soc. Sci. **6**, 433-449. (10.1080/21582041.2011.619347)

[16] Küpper M, Seyfried A. 2020 Analysis of space usage on train station platforms based on trajectory data. Sustainability **12**, 8325. (10.3390/su12208325)

[17] Goffman E. 1971 Relations in public. Microstudies of the public order. New York, NY: Basic Books.

[18] Goffman E. 1966 Behavior in public places. New York, NY: Free Press.

[19] Goffman E. 1989 Interaction ritual. Essays on face-to-face behavior. New York, NY: Pantheon Books.

[20] Collins R. 2014 Interaction ritual chains. Princeton Studies in Cultural Sociology. Princeton, NJ: Princeton University Press.

[21] Waldenfels B. 2015 Sozialität und alterität. Modi sozialer erfahrung. Suhrkamp taschenbuch wissenschaft, vol. 2137, erste auflage, originalausgabe. Berlin, Germany: Suhrkamp.

[22] Postmes T, Haslam SA, Swaab RI. 2005 Social influence in small groups: an interactive model of social identity formation. Eur. Rev. Soc. Psychol. **16**, 1-42. (10.1080/10463280440000062)

[23] Postmes T, Spears R, Lee AT, Novak RJ. 2005 Individuality and social influence in groups: inductive and deductive routes to group identity. J. Pers. Soc. Psychol. **89**, 747-763. (10.1037/0022-3514.89.5.747) 16351366

[24] Jans L, Postmes T, van der Zee KI. 2011 The induction of shared identity: the positive role of individual distinctiveness for groups. Pers. Soc. Psychol. Bull. **37**, 1130-1141. (10.1177/0146167211407342) 21525328

[25] Broekman A vM, Koudenburg N, Gordijn EH, Krans KLS, Postmes T. 2019 The impact of art: exploring the social-psychological pathways that connect audiences to live performances. J. Pers. Soc. Psychol. **116**, 942-965. (10.1037/pspi0000159) 30667257

[26] Schütz A, Luckmann T. 2017 Strukturen der Lebenswelt, 2nd edn. Stuttgart, Germany: UVK Verlag.

[27] Knoblauch H. 2009 Social constructivism and the three levels of video analysis. In Video interaction analysis: methods and methodology (ed. UT Kissmann), pp. 181-198. Berlin, Germany: Peter Lang Publishing.

[28] Knoblauch H, Tuma R. 2014 Videography: an interpretative approach to video-recorded micro-social interaction. In The SAGE handbook of visual research methods (eds E Margolis, L Pauwels), pp. 414-430. Los Angeles, CA: SAGE.

[29] Boltes M. 2015 Automatische Erfassung präziser Trajektorien in Personenströmen hoher Dichte. Jülich, Germany: Forschungszentrum Jülich, Zentralbibliothek.

[30] Drury J. 2009 The nature of collective resilience: survivor reactions to the 2005 London Bombings. Int. J. Mass Emergencies Disasters **27**, 66-95. (10.1177/028072700902700104)

[31] Dezecache G, Martin J-R, Tessier C, Safra L, Pitron V, Nuss P, Grèzes J. 2021 Nature and determinants of social actions during a mass shooting. PLoS ONE **16**, e0260392. (10.1371/journal.pone.0260392) 34874974 PMC8651140

[32] Gérin-Lajoie M, Richards CL, McFadyen BJ. 2005 The negotiation of stationary and moving obstructions during walking: anticipatory locomotor adaptations and preservation of personal space. Motor Control **9**, 242-269. (10.1123/mcj.9.3.242) 16239715

[33] Murakami H, Feliciani C, Nishiyama Y, Nishinari K. 2021 Mutual anticipation can contribute to self-organization in human crowds. Sci. Adv. **7**, eabe7758. (10.1126/sciadv.abe7758) 33731351 PMC7968841

[34] Adrian J, Seyfried A, Sieben A. 2020 Crowds in front of bottlenecks at entrances from the perspective of physics and social psychology. J. R. Soc. Interface **17**, 20190871. (10.1098/rsif.2019.0871) 32343932 PMC7211470

[35] Ezaki T, Ohtsuka K, Chraibi M, Boltes M, Yanagisawa D, Seyfried A, Schadschneider A, Nishinari K. 2016 Inflow process of pedestrians to a confined space. Coll. Dyn. **1**, 1-18. (10.17815/CD.2016.4)

[36] Chraibi M, Tordeux A, Schadschneider A, Seyfried A. 2019 Modelling of pedestrian and evacuation dynamics. In Complex dynamics of traffic management (ed. BS Kerner), pp. 1-22. Berlin, Germany: Springer.

[37] Seitz MJ, Bode NWF, Köster G. 2016 How cognitive heuristics can explain social interactions in spatial movement. J. R. Soc. Interface **13**, 20160439. (10.1098/rsif.2016.0439) 27581483 PMC5014069

[38] Konya K, Sieben A. Waiting and walking with strangers: a socio-psychological pedestrian experiment on joint action in anonymous situations. Pedestrian Dynamics Data Archive. (10.34735/ped.2020.2) PMC10245200 37293361

[39] Konya K, Sieben A. 2023 Waiting and walking with strangers: a socio-psychological pedestrian experiment on joint action in anonymous situations. Figshare. (10.6084/m9.figshare.c.6662754) PMC10245200 37293361

